# Gastric glomus tumor presenting with massive upper GI bleeding: A challenging to diagnose and treat tumor

**DOI:** 10.1002/ccr3.6172

**Published:** 2022-08-03

**Authors:** Ahmad Ayash, Nasrein Elkomy, Muneera Jassim Al‐Mohannadi, Saad Rashid Al Kaabi, Mahir Petkar

**Affiliations:** ^1^ Department of Gastroenterology Hamad Medical Corporation Doha Qatar; ^2^ Department of Pathology Hamad Medical Corporation Doha Qatar

**Keywords:** gastric subepithelial lesions, GI bleeding, glomus tumor

## Abstract

Glomus tumors are infrequent benign tumors that originate from the glomus body and can be found anywhere in the body including the gastrointestinal tract. It is rare that gastric glomus tumors present with life‐threatening upper GI bleeding. Diagnosis of gastric glomus tumors poses a challenge due to overlapping endoscopic and endosonographic features with other gastric subepithelial lesions, and the final diagnosis may not be clear until after surgical resection and pathological examination. We report the case of a 61‐year‐old patient who presented with massive upper GI bleeding secondary to an ulcerated gastric glomus tumor that was eventually treated with surgical wedge resection of the tumor.

## Graphical abstract

Although very rare, it is possible that gastric glomus tumors present with life‐threatening GI bleeding. It should be considered in the differential diagnosis of gastric subepithelial lesions, and the final diagnosis may not be clear until after surgical resection and pathological examination.

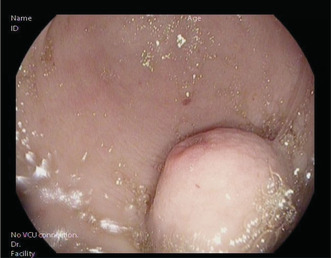

## 1 INTRODUCTION

Glomus tumors (GTs) are rare benign tumors arising from modified smooth muscle cells of the glomus body.^1^ GTs usually occur in the distal extremities, particularly in the nail beds of the fingers. However, they can occur anywhere in the body, including in the gastrointestinal (GI) tract, and most GI tumors are located in the gastric antrum.^1^ Gastric glomus tumors (GGTs) are a rare cause of upper GI bleeding. GGTs usually present a wide variety of symptoms, such as hematemesis, melena, and dyspepsia, but they are sometimes asymptomatic and discovered incidentally.^2^

We report a case of GGT that presented with life‐threatening upper GI bleeding, which was initially treated endoscopically and then ultimately surgically resected as a definitive treatment.

## 2 CASE PRESENTATION

The case was a 61‐year‐old patient. The significant aspects of his medical history were as follows: type 2 diabetes mellitus, hypertension, end‐stage renal disease on regular hemodialysis, coronary artery disease, and rectal cancer status post‐abdominoperineal resection with permanent colostomy.

He was referred to the endoscopy unit by his oncologist for a workup of iron deficiency anemia, with a history of passing dark stools intermittently for the past few weeks.

On clinical examination, the patient was hemodynamically stable. There was pallor with a normal abdominal examination. There was no evidence of melena at the time of examination. Laboratory tests revealed: hemoglobin = 6.4 g/dl, platelet count = 357,000/μl, INR = 1, urea = 23.5 mmol/L, and creatinine = 342 μmol/L. Liver function test results were normal.

**FIGURE 1:**
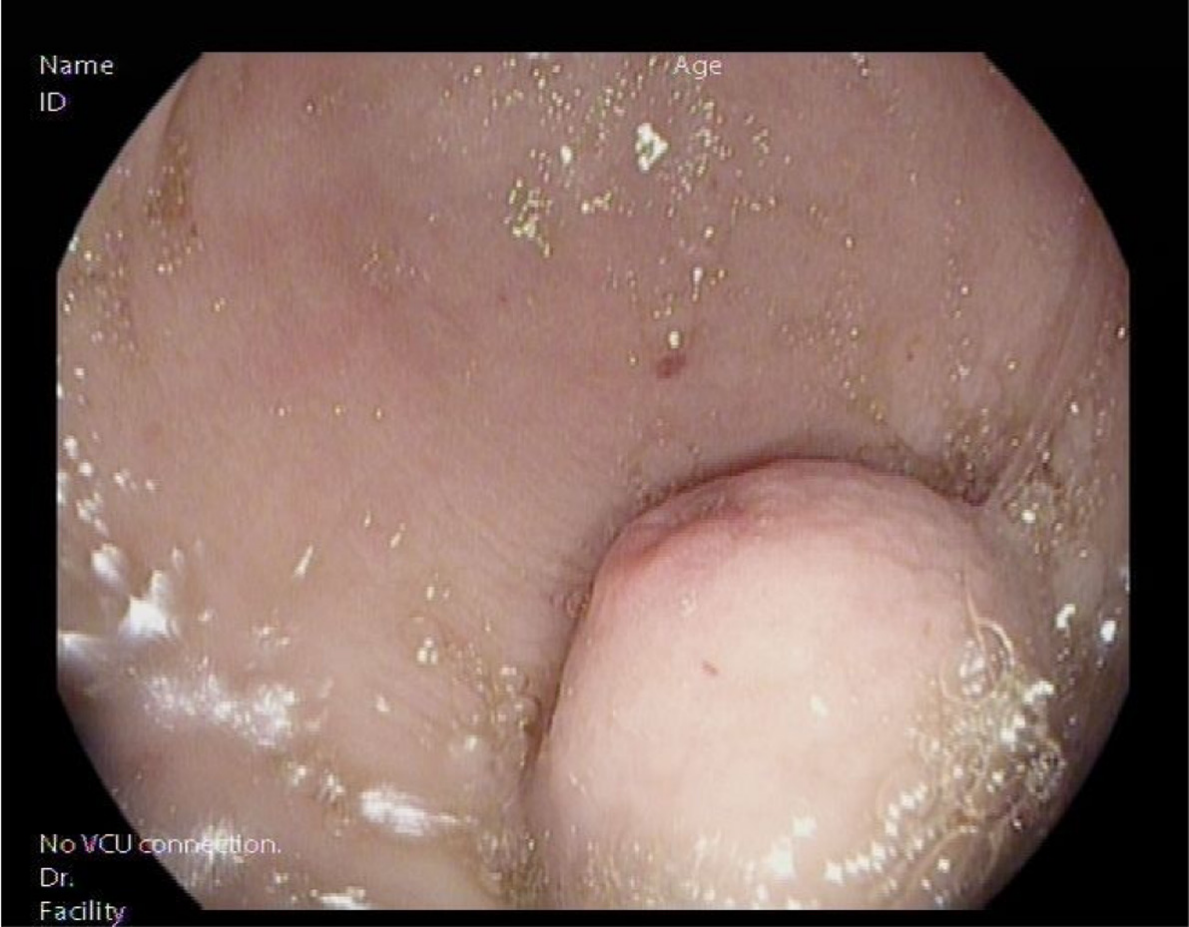
Endoscopic image of the gastric glomus tumor

Esophagogastroduodenoscopy (EGD) revealed an approximately 3 cm subepithelial lesion in the antrum with a clean‐based ulcer in the center. There was no evidence of altered or fresh blood in the stomach. A colonoscopy was performed through the colostomy and was normal (See Figure 1).

**FIGURE 2:**
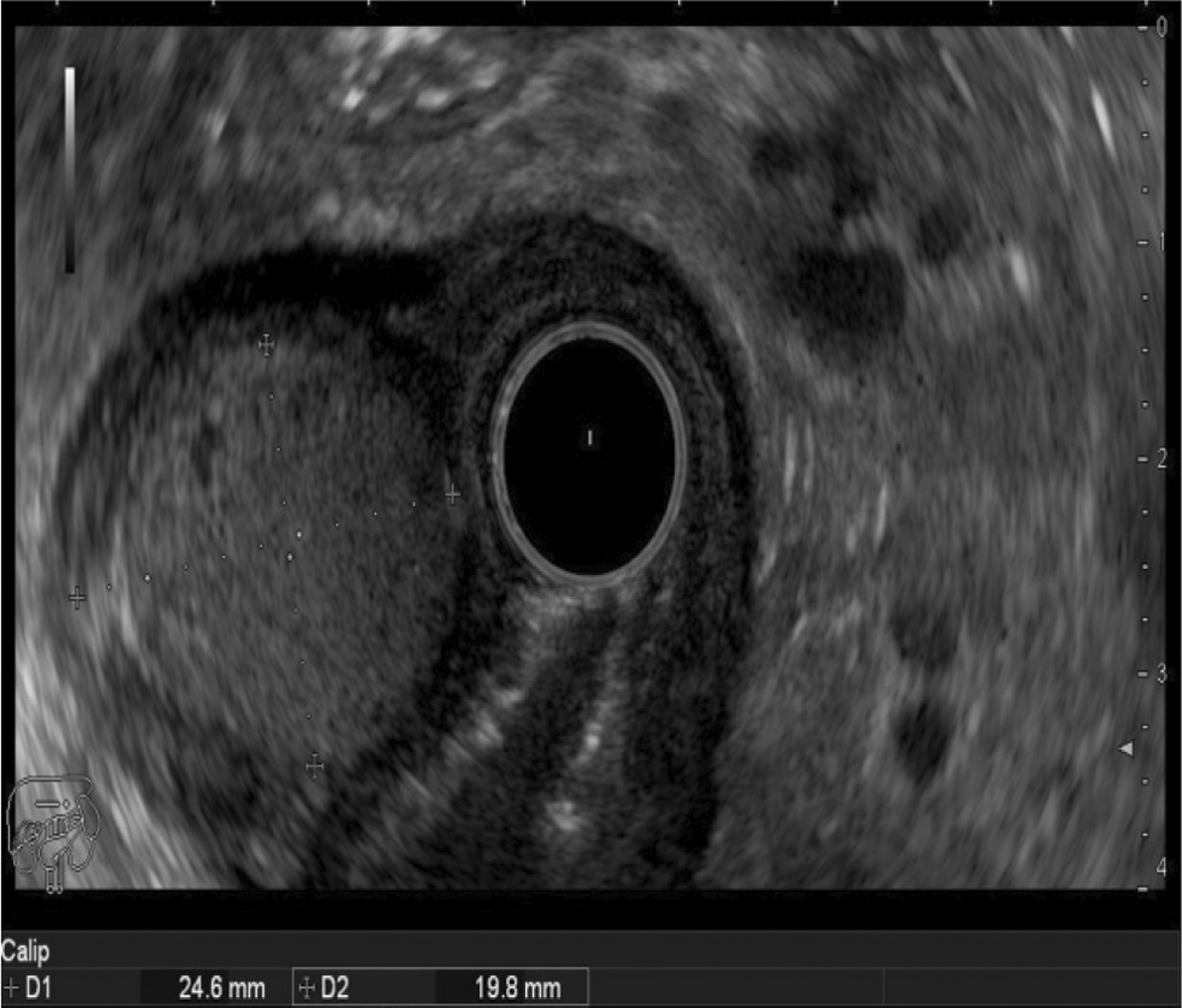
EUS image of the tumor arising from the 2nd and 3rd layer

Subsequently, an endoscopic ultrasound (EUS) was conducted and revealed a hyperechoic antral lesion originating from the second and third layer. The EUS findings were suggestive of lipoma (See Figure 2).

**FIGURE 3:**
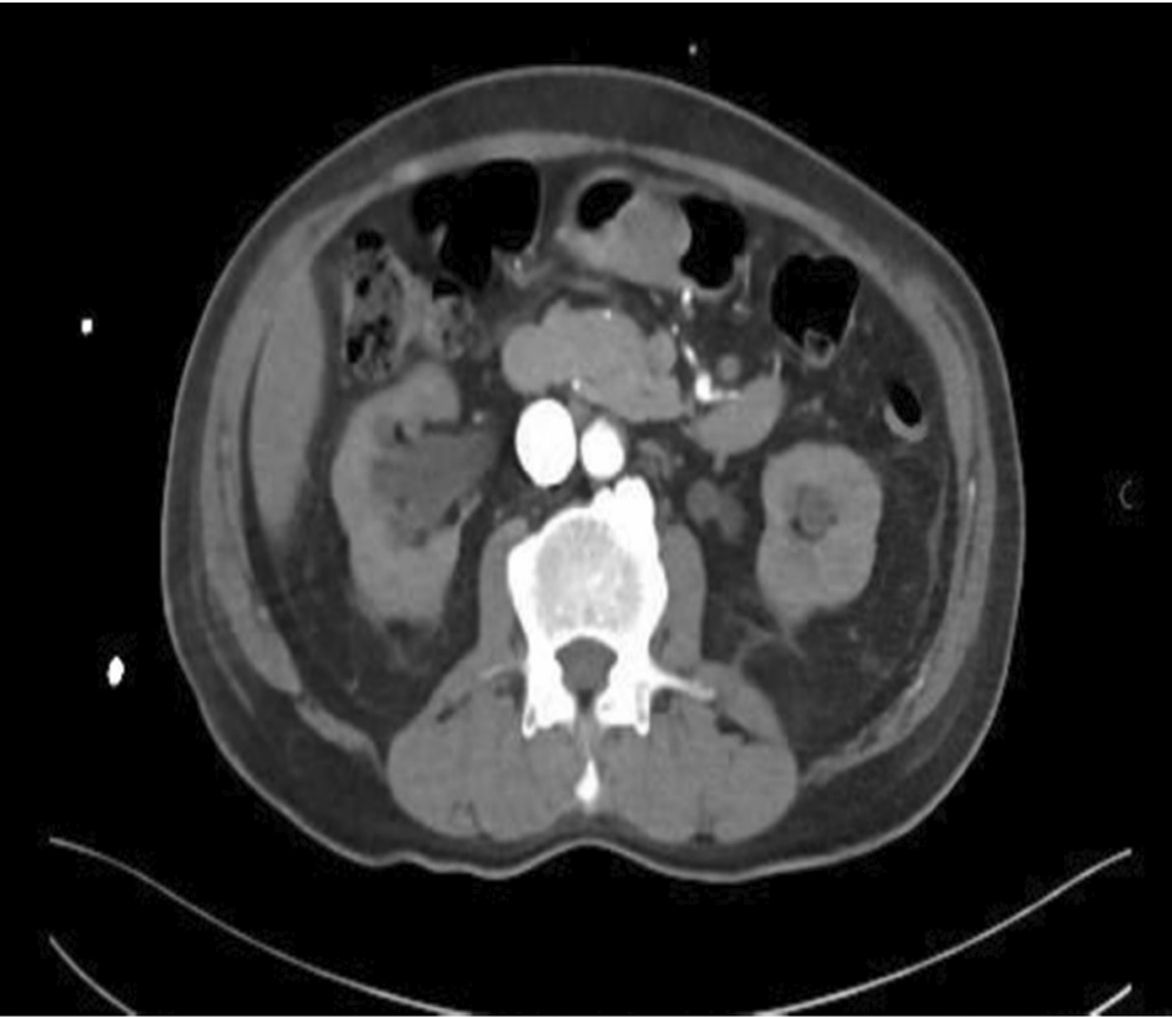
Abdomen CT scan with contrast (Axial image) shows the subepithelial tumor in the gastric antrum.

An abdominal CT scan with contrast was performed. The images showed a well‐defined subepithelial soft tissue mass lesion in the antrum measuring 3 cm and bulging in the stomach lumen. The lesion showed heterogeneous post‐contrast enhancement. There was no definite invasion of the adjacent structures (See Figure 3). The upper GI surgeon and the gastroenterologist held a multidisciplinary team meeting. They agreed to observe the patient and treat him conservatively.

Six weeks later, the patient presented to the emergency department with a 1‐day history of dizziness and hematemesis. The patient deteriorated rapidly in the emergency department within a few hours. On clinical examination, he was drowsy and hemodynamically unstable, with signs of shock. His blood pressure was 84/52 mmHg, heart rate was 100 BPM, respiratory rate was 29 breaths/min, and O2 saturation was 90% on room air. The colostomy bag contained melena.

Laboratory tests revealed a hemoglobin level of 3.2 g/dl, a platelet count of 155,000/μl, an INR of 1.2, urea at 46 mmol/L, and creatinine at 511 μmol/L. He was intubated and admitted to the medical intensive care unit as a case of upper GI bleeding with hemorrhagic shock. He received a packed red blood cell transfusion and was started on an intravenous pantoprazole infusion. An urgent bedside EGD was performed and revealed that the previously observed antral subepithelial lesion had blood oozing from the ulcer at the tip of the lesion. Two endoclips were deployed, and hemostasis was achieved.

To prevent further life‐threatening GI bleeding, the decision was made to surgically remove the lesion.

After an explanation of the benefits and risks of the surgery, the patient agreed to proceed with the procedure. Subsequently, he underwent open wedge resection of the stomach. A midline laparotomy wound was opened from the xiphoid process to the umbilicus, then dissection of omental adhesions was carried out. Gastric mass was identified at greater curvature, and wedge resection was carried with stapler Echelon.

**FIGURE 4:**
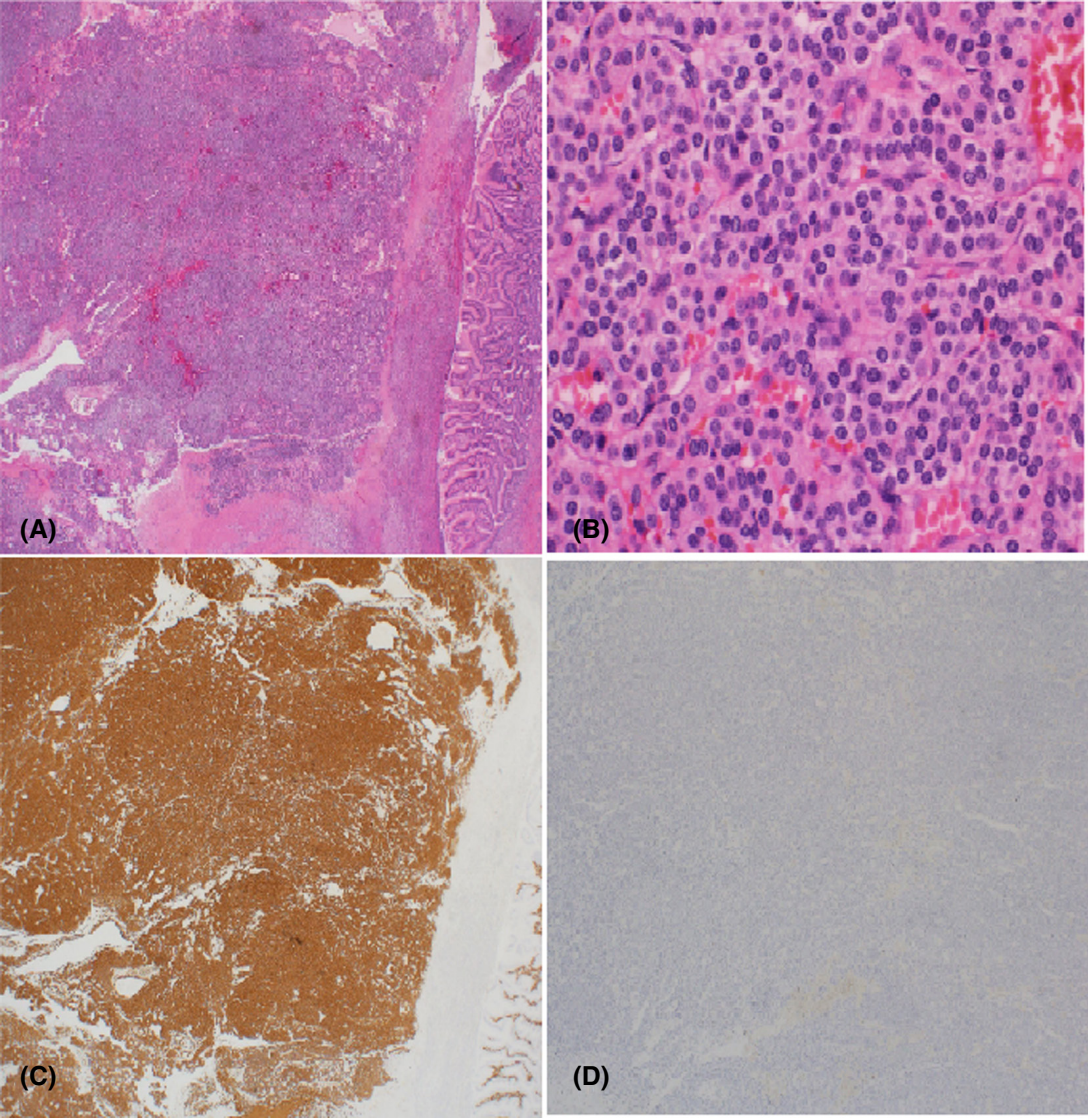
(A–D) Histopathology features of the tumor, (A) Well circumscribed glomus tumor, located in the wall of the stomach (H and E ×2), (B) Higher power view showing characteristic nuclear and cytoplasmic features of glomus cells (H and E ×20), (C) The tumor is strongly positive for S100, (D) Desmin immunostain is negative.

Histopathologic examination of the gastric mass showed a well‐circumscribed 3 cm tumor composed of cohesive clusters of glomus cells containing round to oval nuclei with pale eosinophilic cytoplasm, infrequent mitotic activity, and low proliferation index of 10%, as measured by Ki‐67 immunohistochemistry. The tumor cells were strongly positive for smooth muscle actin, calponin, caldesmon, and synaptophysin, and negative for CD 117, CD 34, CD 56, S 100, CK 20, and chromogranin. Excision was complete. The morphological and immunohistochemical features confirmed the diagnosis of a glomus tumor (See Figure 4A–D).

The patient was doing well after 9 months of follow‐up post‐surgery. There was no evidence of GI bleeding again and his hemoglobin returned to baseline levels.

## 3 DISCUSSION

Glomus tumors are rare mesenchymal tumors arising from the neuromyoarterial glomus. They account for 1% of gastrointestinal stromal tumors (GISTs).^3^ Despite being reported in the literature in early 1951 by Kay et al.,^4^ few cases have been reported since, and there are no established guidelines for their management. Most GGTs are solitary and located in the gastric antrum; they are rarely reported in multiples.^5^ Moreover, they might present with abdominal pain, gastrointestinal bleeding, or perforation.^6^ It can also be found incidentally, with no symptoms.^7^ Although GGTs have distinctive macroscopic and microscopic morphology, they have some overlapping features with other GISTs and carcinoid tumors.^8^ This case demonstrates that the diagnosis of GGT poses a challenge, given that it lacks typical endoscopic and clinical features and shares many features with other gastric subepithelial tumors. Upper GI bleeding is known to be associated with GGTs; However, it has not been reported to be life‐threatening or to happen in non‐ulcerated GGTs.

In CT scans, GGTs appear as well‐circumscribed subepithelial masses that have strong arterial phase enhancement, a progressive filled‐in enhanced pattern, and prolonged enhancement during multiphasic scans. Unfortunately, CT imaging cannot differentiate between GGTs and other mesenchymal tumors.^9^ For a definite diagnosis, EUS‐FNB with immunohistochemical examination must be performed. In EUS, GGTs appear as hypoechoic, well‐circumscribed lesions in the submucosa and/or muscularis propria layers.^10^ Pathological examination of GGTs shows small uniform epithelioid cells that are positive for smooth muscle actin and calponin and exhibit PAS‐positive basement membranes. The cells are negative for desmin, cytokeratin (AE1/AE3b), S‐100, C‐KIT (CD‐117), CD34, DOG1, chromogranin A, p53, and neuron‐specific enolase.^11^, ^12^

The treatment of choice for GGT is surgery with negative margins, which may include resection of the tumor, subtotal gastrectomy, or wedge resection.^13^ The laparoscopic approach is preferred over open surgery because of decreased risk of postoperative complications and morbidity.^14^

## 4 CONCLUSION

In this case report, GGT was initially misdiagnosed as a lipoma and later presented with a life‐threatening GI bleed that was ultimately treated with surgical resection of the tumor. GGT is a rare disease with limited published knowledge, and there are no definitive guidelines for diagnosis and treatment. To date, the course of GGT management has been left to the physician's discretion.

## AUTHOR CONTRIBUTIONS

Ahmad Ayash and Nasrein Elkomy reviewed the literature, drafted and edited the manuscript, and approved the final manuscript. Muneera Jassim Al‐Mohannadi, Saad Rashid Al‐Kaabi, and Mahir Petkar reviewed the literature and edited and approved the final manuscript. The manuscript was prepared according to ICJME guidelines and CARE guidelines for reporting case reports.

## CONFLICT OF INTEREST

The authors declare that they have no conflicts of interest.

## ETHICAL APPROVAL

The case report was approved (MRC‐04‐21‐641) by the IRB and Medical Research Centre of Hamad Medical Corporation, Doha, Qatar.

## CONSENT

Written informed consent was obtained from the patient to publish this report in accordance with the journal's patient consent policy.

## Data Availability

Data sharing not applicable to this article as no datasets were generated or analyzed during the current study
